# Accelerometer-Based Physical Activity and Health-Related Quality of Life in Korean Adults: Observational Study Using the Korea National Health and Nutrition Examination Survey

**DOI:** 10.2196/59659

**Published:** 2024-09-03

**Authors:** Sujeong Han, Bumjo Oh, Ho Jun Kim, Seo Eun Hwang, Jong Seung Kim

**Affiliations:** 1 Department of Family Medicine SMG-SNU Boramae Medical Center Seoul Republic of Korea; 2 College of Medicine Seoul National University Seoul Republic of Korea; 3 Department of Family Medicine Hongseoung Medical Center Chungcheongnam-do Republic of Korea; 4 Department of Family Medicine Seoul National University Hospital Seoul Republic of Korea

**Keywords:** Health-Related Quality of Life (HRQoL), physical activity, Accelerometer, Korea National Health and Nutrition Examination Survey (KNHANES), mobile phone

## Abstract

**Background:**

Health-related quality of life (HRQoL) reflects an individual's perception of their physical and mental health over time. Despite numerous studies linking physical activity to improved HRQoL, most rely on self-reported data, limiting the accuracy and generalizability of findings. This study leverages objective accelerometer data to explore the association between physical activity and HRQoL in Korean adults.

**Objective:**

The objective of this study is to analyze the relationship between objectively measured physical activity using accelerometers and HRQoL among Korean adults, aiming to inform targeted interventions for enhancing HRQoL through physical activity.

**Methods:**

This observational study included 1298 participants aged 19-64 years from the Korea National Health and Nutrition Examination Survey (KNHANES) VI, who wore an accelerometer for 7 consecutive days. HRQoL was assessed using the EQ-5D questionnaire, and physical activity was quantified as moderate-to-vigorous physical activity accelerometer-total (MVPA-AT) and accelerometer-bout (MVPA-AB). Data were analyzed using logistic regression to determine the odds ratio (ORs) for low HRQoL, adjusting for socioeconomic variables and mental health factors.

**Results:**

Participants with higher HRQoL were younger, more likely to be male, single, highly educated, employed in white-collar jobs, and had higher household incomes. They also reported less stress and better subjective health status. The high HRQoL group had significantly more participants meeting MVPA-AB ≥600 metabolic equivalents (*P*<.01). Logistic regression showed that participants meeting MVPA-AB ≥600 metabolic equivalents had higher odds of high HRQoL (OR 1.55, 95% CI 1.11-2.17). Adjusted models showed consistent results, although the association weakened when adjusting for mental health factors (OR 1.45, 95% CI 1.01-2.09).

**Conclusions:**

The study demonstrates a significant association between HRQoL and moderate to vigorous physical activity sustained for at least 10 minutes, as measured by accelerometer. These findings support promoting physical activity, particularly sustained moderate to vigorous activity, to enhance HRQoL. Further interventional studies focusing on specific physical activity domains such as occupational, leisure-time, and commuting activities are warranted.

## Introduction

Physical activity (PA) is defined as any bodily movement produced by skeletal muscles that results in energy expenditure [1]. It includes all activities such as work, housework, commuting, and leisure. PA is widely recognized as a critical component of healthy lifestyle, contributing significantly to the prevention and management of various chronic diseases such as cardiovascular disease, diabetes, and obesity [2-4]. Despite various guidelines for PA based on many studies, the rate of aerobic PA in Korea has been continuously decreasing, with the rate among women being less than half (44%) [5].

Quality of life (QoL) is defined as an individual’s perception of their position in life in the context of the culture and value systems where they live and in relation to their goals, expectations, standards, and concerns [6]. It is divided into health-related quality of life (HRQoL) and non-HRQoL [7]. As medical technology and accessibility advances, life expectancy increases, and major health problems become chronic diseases; not only the interest in treatment but also the management and prevention of diseases is increasing. HRQoL is a concept used to help determine an individual's physical and mental health to help prevent disease and make treatment decisions [8]. It is important because it can be applied to actual clinical treatment through research.

Previous studies have shown a positive association between PA and HRQoL, emphasizing the importance of maintaining active lifestyle for overall well-being. For instance, a study by Scarabottolo et al [9] found that different domains of PA (occupational PA and leisure-time sports practice) were significantly associated with improved HRQoL. Similarly, Puciato et al [10] reported that PA positively influenced the QoL in working-age people in Poland. However, most of the studies have predominantly focused on elderly populations or patients with specific diseases [11-15]. Furthermore, the majority of these studies have relied on self-reported questionnaires to measure PA [9,10,13,15-17].

In recent years, the use of accelerometers to objectively measure PA levels has gained popularity, offering more accurate and reliable data compared to self-reported measures [18-21]. This advancement provides deeper insights into the relationship between PA and various health outcomes. However, studies investigating the association between objectively measured PA using devices such as accelerometers and HRQoL are relatively scarce, especially in Korea.

Therefore, the aim of this study was to analyze the association between objective PA, as measured by accelerometers, and HRQoL in Korean adults using data from the Korea National Health and Nutrition Examination Survey (KNHANES). By analyzing this relationship, we anticipate to contribute to the development of evidence-based strategies to promote PA and enhance HRQoL.

## Methods

### Data Source and Study Population

This study was based on data from the sixth KNHANES (KNHANES VI) conducted from 2014 to 2015 by the Korea Centers for Disease Control and Prevention (KCDC). KNHANES is a legal survey conducted annually on the level of health of the public, health-related awareness and behaviors, chronic diseases, and food and nutrition intake.

**Figure 1:**
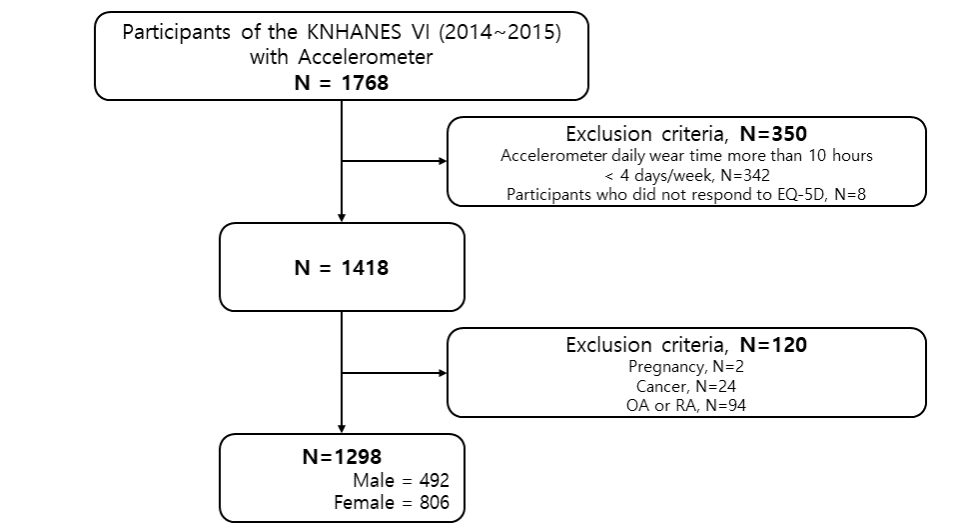
Flowchart of the study populations. KNHANES: Korea National Health and Nutrition Examination Survey; OA: Osteoarthritis; RA: Rheumatoid arthritis.

Among the participants of the KNHANES VI (2014-2015), 1827 people participated in the accelerometer survey, but 59 people were excluded due to loss of the accelerometer (9 people), nonwearers (47 people), and mechanical errors (3 people). A total of 1418 people were selected first, excluding 342 people who did not meet the minimum wearing time and number of days of the accelerometer used in the previous study for more than 10 hours a day, 4 days a week, and 8 people who did not respond to the health-related quality of life survey (EQ-5D). Among them, 1298 people were selected as participants for final analysis, excluding 2 pregnant women, 24 people receiving cancer treatment, and 94 people with osteoarthritis or rheumatoid arthritis that could affect their PA (Figure 1).

### Ethical Considerations

It was approved by the Research Ethics Review Committee of the KCDC (Approval No. 2013-12 EXP-03-5C, 2015-01-02-6C).

### Variables and Measurements: Health-Related Quality of Life

Tools to evaluate HRQoL include World Health Organization Quality of Life Brief Version (WHOQOL-BREF) [6], Quality of Well-Being Scale [22], 36-Item Short Form [23], and EQ-5D [24]. Among them, the reliability and validity of the Korean version of EQ-5D reliability and validity have been verified in several studies [25,26].

EQ-5D, a self-report questionnaire, was used for measurement of HRQoL. The survey questions were answered on a three-step scale (no problem, moderate problem, severe problem) with 5 questions: mobility, self-care, usual activity, pain/disability, and anxiety/depression. The KCDC calculated the EQ-5D index using the following formula to express it as a single index.

EQ-5D index = 1- (0.050+0.096×M2+0.418×M3+0.046×SC2+0.136×SC3+0.051×UA2 +0.208×UA3+0.037×PD2+0.151×PD3+0.043×AD2+ 0.158×AD3+0.050×N3)

Based on previous research methods [8], the EQ-5D index was divided into 2 groups, below average (low quality of life, low QoL) and above average (high quality of life, high QoL).

### PA

#### PA Measured by Accelerometer

The accelerometer used in KNHANES is the wGT3X+ (ActiGraph LLC), and it is a device that converts the acceleration of movement displayed by PA into an electrical signal. The accelerometer and a written consent form were provided to adults 19-64 years old who consented to wear the accelerometer. Measurements were programmed to record from midnight (midnight) on the next day after delivery, and they were instructed to wear the accelerometer on the waist for 7 consecutive days except for swimming, showering, and sleeping.

The following criteria were applied with reference to previous studies [18,27]: (1) data summary cycle (60 seconds); (2) intensity of PA (count per minute [CPM], sedentary behavior <100; 2000 ≤moderate PA ≤5998; vigorous PA ≥5999); (3) determination algorithm of accelerometer wearing or nonwearing time (If CPM is 0 and lasts longer than 60 minutes, it is considered nonwearing time. However, if CPM is less than 100 and lasts less than 2 minutes, it is acceptable); (4) accelerometer minimum wearing time and days (10 hours per day, 4 days per week); and (5) criteria for meeting PA guidelines (600 metabolic equivalent [MET]-minutes/week: [(moderate PA × 4 METs) + (vigorous PA × 8 METs)] ≥ 600 METs)

PA measured with an accelerometer was quantified in two ways: (1) Moderate to vigorous PA accelerometer-total (MVPA-AT, for at least 1 minute), and (2) Moderate to vigorous PA accelerometer-bout (MVPA-AB, for at least 10 minutes; if the time when the corresponding strength number of cutting points has not been reached is less than 2 minutes, it is acceptable). One minute of vigorous PA was counted as 2 minutes of moderate PA.

#### Self-Reported PA

Self-reported PA was collected by using the Global Physical Activity Questionnaire (GPAQ), divided into 3 categories: leisure, occupation, and commuting PA. Since the PA measured with the accelerometer was collected for most of the time while wearing the accelerometer, all 3 physical activities (leisure, occupation, comminuting) were summed up. After that, the same criteria for the PA measured by accelerometer were applied.

#### Covariates

Socioeconomic factors such as age, sex, marital status, education level, employment, household income, and residence were investigated. Employment was classified according to occupational reclassification and unemployment and economic inactivity status codes. After that, it was divided into 3 groups: white collar (managers, experts, related workers, and office workers), blue collar (service/sales workers, skilled workers in agriculture, forestry and fisheries, craftsmen, workers in machine operation/assembly, and simple labor workers), and unemployed (housewives, students, etc). Household income level was classified into 2 groups: the bottom 50% (Low) and the top 50% (High) of household income. Residence was classified into *dong* (urban) and *eup*/*myeon* (rural).

Health-related lifestyle factors including smoking, drinking, and average daily sleep time were investigated. Smoking was classified into 3 groups: nonsmoker (person who never smoked or smoked less than 5 packs or 100 cigarettes in their lifetime), past smoker (person who smoked in the past but not now), and smoker (person who currently smokes). Alcohol use was classified into 3 groups according to the WHO high-risk drinking standards: heavy drinker (14 or more drinks per week for men/10 or more drinks per week for women), adequate drinker (annual drinker, not heavy drinker), and abstainer (those who have not drunk alcohol in their lifetime).

Mental health factors including stress perception rate and subjective health status were investigated. Stress was classified into 2 categories with answers to the question “How much stress do you usually feel in your daily life?”: Stressful (I feel a lot, I feel a little), little stress (I hardly feel it). Subjective health status was classified into 3 categories with answers to the question “How do you usually feel about your health?”: good (very good, good), normal (average), and poor (bad, very bad).

Variables related to chronic disease, the prevalence of cardiovascular disease, diabetes, and depression was investigated. Cardiovascular disease includes stroke, angina, and myocardial infarction. The prevalence of each disease was classified into those diagnosed by a doctor.

### Statistical Analysis

Data are presented as the mean (SD) for continuous variables, and presented as number and percent for categorical variables. We analyzed the study participants’ characteristics according to the EQ-5D index, using *t* test to compare continuous variables, chi-square test for categorical variables.

Additionally, adjusted odds ratio (OR) and 95% CIs for the risk of low HRQoL according to PA measured by the accelerometer and GPAQ were calculated using logistic regression after adjusting the socioeconomic variables (age, sex, marital status, education, employment, and income) for model 1, covariates in model 1 plus mental health-related variables (stress, subjective health status, and depression) for model 2.

All statistical analyses were performed using STATA (version 18.0; StataCorp) and *P* values of <.05 were considered to indicate statistical significance.

## Results

### General Characteristics

Table 1 shows the baseline characteristics of the below-average (low QoL) and above-average (high QoL) groups of the EQ-5D index. Age was significantly lower in the high QoL group. In addition, males, singles, highly educated people, office workers, and high-income earners were more common in the high QoL group. Those who usually feel less stress and those whose subjective health was good were more common in the high QoL group. There was no significant difference between the 2 groups in some health-related lifestyle variables such as smoking, drinking, and average sleep time. Among chronic disease-related variables, cardiovascular disease and diabetes were not significantly different between the 2 groups.

**Table 1:** Baseline characteristics of the study population by quality of life.

Variable		Low QoL^a^ (n=293)	High QoL (n=1005)	*P* value^b^	
Age in years, mean (SD)		45.43 (11.72)	43.18 (12.34)	.006	
**Sex, n (%)**					<.001
	Male	84 (28.67)	408 (40.60)		
	Female	209 (71.33)	597 (59.40)		
**Marital status, n (%)**					<.001
	Single	46 (15.70)	216 (21.51)		
	Married	216 (73.72)	740 (73.71)		
	Separated, divorced, or widowed	31 (10.58)	48 (4.78)		
**Education, n (%)**					<.001
	Middle school or less	67 (22.08)	139 (13.83)		
	High school	119 (40.61)	410 (40.80)		
	College or more	107 (36.52)	456 (45.37)		
**Employment, n (%)**					.003
	White collar	68 (23.29)	339 (33.80)		
	Blue collar	118 (40.41)	361 (35.99)		
	Unemployed	106 (36.30)	303 (30.21)		
**Household income, n (%)**					.009
	Low	110 (37.54)	296 (29.51)		
	High	183 (62.46)	707 (70.49)		
**Residence, n (%)**					.45
	Urban	237 (80.89)	832 (82.79)		
	Rural	56 (19.11)	173 (17.21)		
**Smoking, n (%)**					.27
	Nonsmoker	218 (74.66)	702 (69.85)		
	Past smoker	38 (13.01)	151 (15.02)		
	Smoker	36 (12.33)	152 (15.12)		
**Alcohol use, n (%)**					.55
	Abstainer	60 (20.55)	234 (23.28)		
	Adequate drinker	197 (67.47)	664 (66.07)		
	Heavy drinker	35 (11.99)	107 (10.65)		
Sleep duration (h), mean (SD)		6.82 (1.33)	6.86 (1.18)	.60	
**Stress, n (%)**					<.001
	Little stressful	176 (60.27)	788 (78.41)		
	Stressful	116 (39.73)	217 (21.59)		
**Subjective health status, n (%)**					<.001
	Poor	89 (30.38)	79 (7.86)		
	Normal	157 (53.58)	545 (54.23)		
	Good	47 (16.04)	381 (37.91)		
**Cardiovascular disease, n (%)**					.91
	No	290 (98.98)	994 (98.91)		
	Yes	3 (1.02)	11 (1.09)		
**Diabetes, n (%)**					.74
	No	281 (95.90)	968 (96.32)		
	Yes	12 (4.10)	37 (3.68)		
**Depression, n (%)**					<.001
	No	267 (91.13)	979 (97.41)		
	Yes	26 (8.87)	26 (2.59)		
Sedentary time (min), mean (SD)		3194.75 (766.72)	3258.27 (764.79)	.21	

^a^QoL: quality of life.

^b^*P* value is from *t* test for continuous variables and Chi-square test for categorical variables.

### PA

Table 2 presents PA measured by the accelerometer and GPAQ in the 2 groups divided by the average of the EQ-5D index. There were significantly more persons who met MVPA-AB ≥600 METs in the high QoL group (*P*<.10). There was no significant difference between high and low QoL groups for MVPA-AT and self-reported PA.

**Table 2:** Comparison of physical activity by health-related quality of life.

Variable			Low QoL^a^ (n=293), n (%)		High QoL (n=1005), n (%)		*P* value^b^
**MVPA^c^-AB^d^ (10 min bouts)**							.01
	<600 METs	243 (82.9)		762 (75.82)			
	≥600 METs	50 (17.1)		243 (24.18)			
**MVPA-AT^e^ (Total bouts)**							.27
	<600 METs^f^	127 (43.3)		400 (39.80)			
	≥600 METs	166 (56.7)		605 (60.20)			
**MVPA-S (Self-reported)**							.73
	<600 METs	127 (43.3)		447 (44.48)			
	≥600 METs	166 (56.7)		558 (55.52)			

^a^QoL: Quality of Life.

^b^*P* values obtained using chi-square tests.

^c^MVPA: moderate to vigorous physical activity.

^d^AB: accelerometer-bout.

^e^AT: accelerometer-total.

^f^METs: metabolic equivalents.

### Association Between the Domains of PA and HRQoL

In Table 3, logistic regression analysis was performed to determine the correlation between HRQoL and PA measured by the accelerometer and GPAQ, and marked with an OR and 95% CI. Compared with the low QoL group, the crude OR of the HRQoL was 1.55 (95% CI 1.11-2.17) in MVPA-AB ≥600 METs. After adjusting for the socioeconomic variables (model 1), the OR of the HRQoL was 1.60 (95% CI 1.13-2.27). After adjusting model 1 for the mental health–related variables (model 2), the OR was 1.45 (95% CI 1.01-2.09), which weakened the statistical significance compared with model 1. There was no statistical significance between the 2 QoL groups for MVPA-AT and self-reported PA.

**Table 3:** Association between domains of physical activity and health-related quality of life.

Domains of physical activity		Number	Crude OR^a^ (95% CI)	Model 1^b^ (95% CI)	Model 2^c^ (95% CI)
**MVPA^d^-AB^e^ (10-min bouts)**					
	<600 METs^f^	1005	1	1	1
	≥600 METs	239	1.55 (1.11-2.17)	1.60 (1.13-2.27)	1.45 (1.01-2.09)
**MVPA-AT^g^ (Total bouts)**					
	<600 METs	527	1	1	1
	≥600 METs	771	1.15 (0.89-1.51)	1.07 (0.82-1.41)	0.91 (0.68-1.22)
**MVPA-S (Self-reported)**					
	<600 METs	574	1	1	1
	≥600 METs	724	0.96 (0.73-1.24)	0.86 (0.66-1.13)	0.79 (0.59-1.06)

^a^OR: odds ratio.

^b^Adjusted for age (continuous), sex, marital status, education, employment, income.

^c^Further adjusted for stress, subjective health status, depression.

^d^MVPA: moderate to vigorous physical activity.

^e^AB: accelerometer-bout.

^f^MET: metabolic equivalent.

^g^AT: accelerometer-total.

## Discussion

### Principle Findings

This study was designed to confirm the relationship between HRQoL and PA measured by an accelerometer using national survey data. As a result of the analysis, HRQoL was significantly associated with the group that engaged in more MVPA for at least 10 minutes, as measured by the accelerometer. However, there was no statistical significance with HRQoL for MVPA measured for at least 1 minute by the accelerometer. In the previous KNHANES, only self-report questionnaires were used to measure PA [8]. However, in the sixth KNHANES (2014-2015), PA measured with an accelerometer was provided, making it possible to conduct studies [28-30] like ours.

Our findings align with previous research demonstrating the beneficial effects of PA on HRQoL across various populations [31,32]. For example, multiple studies have reported that increased PA is associated with improved HRQoL. A study by Brown et al [16] found that adults who engaged in regular MVPA had significantly higher HRQoL scores compared to those who were inactive. Similarly, a review by Marquez et al [33] synthesized evidence from numerous studies and concluded that PA interventions can effectively enhance HRQoL across various populations. PA has been shown to enhance self-efficacy, physical self-esteem, and positive affect [32,34], which collectively contribute to improvements in HRQoL. Overall, our results are consistent with the majority of previous literature on the positive relationship between PA and HRQoL. This study further supports these findings by using objective measures of PA, thus providing a more accurate assessment of its impact on HRQoL.

This study can serve as a basis for recommending at least 10 minutes of MVPA to improve HRQoL. However, the phrase “at least 10 minutes” was deleted from the PA guidelines published by the US Department of Health and Human Services in 2018, as MVPA for less than 10 minutes can benefit health. Therefore, to compare this with other health-related variables not considered in this study, it is necessary to measure MVPA for at least 1 minute. Nevertheless, since the PA questionnaire used in the KNHANES specifies “usually continued PA for at least 10 minutes during a week,” it is more appropriate to compare it with PA for more than 10 minutes, as measured with an accelerometer in this study [35].

Earlier, it was mentioned that the GPAQ calculates PA that has been continued for at least 10 minutes, so it is appropriate to compare it with PA measured with an accelerometer for at least 10 minutes. However, previous studies [28,36-38] found no correlation between accelerometer data and the questionnaire's satisfaction with the PA guidelines, and the results were not consistent in this study as well. This inconsistency may arise because the questionnaire’s criteria for “PA continued for at least 10 minutes” are subjective or may include PA lasting less than 10 minutes. Additionally, there is a possibility of bias in the self-report questionnaire, as people engaging in MVPA may over-report their activity levels [37-39]. Furthermore, since the accelerometer was provided after filling out the questionnaire, the data measurement periods differ, and the GPAQ may misclassify location movement PA as MVPA without considering movement speed. Therefore, to measure PA more accurately, a specific method that can complement both approaches is necessary.

In previous studies [40-42], stress and subjective health status perception had a negative correlation with HRQoL, and in 1 study [43]; it was reported that stress had the greatest influence on the deterioration of women's QoL. In this study, when socioeconomic variables were corrected for the group with a lot of PA measured with an accelerometer, the statistical significance was stronger than before the correction. But when stress and subjective health status perception were additionally corrected, the degree of statistical significance decreased. Similar to previous studies, this can be interpreted as having a greater effect on stress and subjective health status perception on HRQoL.

### Limitations

This study has the following limitations. First, it is difficult to generalize the study results since it is a nonprobability sample composed of study subjects selected through convenience sampling. Second, variables including EQ-5D are investigated through a self-report questionnaire, so there is a possibility of social desirability bias, misclassification bias, and recall bias. Third, since it was conducted as an observational cross-sectional study, it is not possible to know the causal relationship between HRQoL and variables. Nevertheless, it is significant that the study was conducted using national health and nutrition survey data of about 1300 adults aged 19-64. Also, since it is a study using data obtained using an accelerometer, it has an advantage in being able to compare it with other domestic and foreign studies conducted in a similar way. Research using accelerometers began to increase in the 2000s, and since the beginning of 2010, nearly 100 related studies have been published annually [44,45]. In addition, the era has come when it is possible to measure PA using wearable devices such as smartphones and smart watches. It is thought that various follow-up studies using tools that can quantify PA are possible in the future. Based on this, we expect the growth of fields related to PA aimed at promoting health.

### Conclusions

Our study shows the association between HRQoL and MVPA, which is significantly higher in the group with more PA for at least 10 minutes, using the KNHANES. The study emphasizes the need for promoting PA and interventions focusing specifically on continuing at least 10 minutes. We expect further interventional studies to focus on the specific PA time period such as occupational PA, leisure-time PA, and commuting PA.

## Abbreviations

CPM: count per minute
GPAQ: Global Physical Activity Questionnaire
HRQoL: health-related quality of life
KCDC: Korea Centers for Disease Control and Prevention
KNHANES: Korea National Health and Nutrition Examination Survey
MET: metabolic equivalent
MVPA-AB: moderate to vigorous physical activity with an accelerometer for at least 10 minutes
MVPA-AT: moderate to vigorous physical activity with an accelerometer for at least 1 minute
MVPA-S: moderate to vigorous physical activity with self-report questionnairePA: physical activity
OR: odds ratio
QoL: quality of life
WHOQOL-BREF: World Health Organization Quality of Life Brief Version
